# KR4SL: knowledge graph reasoning for explainable prediction of synthetic lethality

**DOI:** 10.1093/bioinformatics/btad261

**Published:** 2023-06-30

**Authors:** Ke Zhang, Min Wu, Yong Liu, Yimiao Feng, Jie Zheng

**Affiliations:** School of Information Science and Technology, ShanghaiTech University, Shanghai 201210, China; Shanghai Institute of Microsystem and Information Technology, Chinese Academy of Sciences, Shanghai 200050, China; University of Chinese Academy of Sciences, Beijing 100049, China; Institute for Infocomm Research, Agency for Science, Technology and Research (A*STAR), Singapore 138632, Singapore; Nanyang Technological University, Singapore 639798, Singapore; School of Information Science and Technology, ShanghaiTech University, Shanghai 201210, China; Lingang Laboratory, Shanghai 201602, China; School of Information Science and Technology, ShanghaiTech University, Shanghai 201210, China; Shanghai Engineering Research Center of Intelligent Vision and Imaging, ShanghaiTech University, Shanghai 201210, China

## Abstract

**Motivation:**

Synthetic lethality (SL) is a promising strategy for anticancer therapy, as inhibiting SL partners of genes with cancer-specific mutations can selectively kill the cancer cells without harming the normal cells. Wet-lab techniques for SL screening have issues like high cost and off-target effects. Computational methods can help address these issues. Previous machine learning methods leverage known SL pairs, and the use of knowledge graphs (KGs) can significantly enhance the prediction performance. However, the subgraph structures of KG have not been fully explored. Besides, most machine learning methods lack interpretability, which is an obstacle for wide applications of machine learning to SL identification.

**Results:**

We present a model named KR4SL to predict SL partners for a given primary gene. It captures the structural semantics of a KG by efficiently constructing and learning from relational digraphs in the KG. To encode the semantic information of the relational digraphs, we fuse textual semantics of entities into propagated messages and enhance the sequential semantics of paths using a recurrent neural network. Moreover, we design an attentive aggregator to identify critical subgraph structures that contribute the most to the SL prediction as explanations. Extensive experiments under different settings show that KR4SL significantly outperforms all the baselines. The explanatory subgraphs for the predicted gene pairs can unveil prediction process and mechanisms underlying synthetic lethality. The improved predictive power and interpretability indicate that deep learning is practically useful for SL-based cancer drug target discovery.

**Availability and implementation:**

The source code is freely available at https://github.com/JieZheng-ShanghaiTech/KR4SL.

## 1 Introduction

Cancer is a group of human diseases for which interactions among many genes play crucial roles (Huang et al. 2020; Jariyal et al. 2020). Therefore, identifying genetic interactions is important for the discovery of anticancer drug targets. Synthetic lethality (SL) is a type of genetic interaction between two genes such that the inhibition of either gene alone leaves cells viable while the inhibition of both genes leads to cell death (Kaelin 2005). This offers a promising strategy of cancer treatment. By targeting a gene that is nonessential in normal cells but synthetic lethal with a gene with cancer-specific alterations, we can kill the cancer cells without harming the normal cells. Besides, while many cancer-driver genes are not directly druggable (e.g. with loss-of-function mutations in tumor cells), we can instead target their SL partner genes, thereby expanding the anticancer drug target space (Setton et al. 2021). Given the advantages of SL-based cancer therapy, extensive efforts have been made to identify SL interactions in cancer cells. Some wet-lab techniques have been developed for large-scale SL screening, such as RNA interference and genome editing with CRISPR/Cas9 (O’Neil et al. 2017). However, the lab-screening techniques face some problems such as high cost and off-target effects (Jariyal et al. 2020). Additionally, so far only a few SL-based drug targets have been validated to be clinically relevant, partly because the mechanisms of many SL pairs identified in the screenings remain unclear (Huang et al. 2020). To address these issues and speed up SL-based drug target discovery, many bioinformatic methods for SL prediction and analysis have been developed over the past decade.

Computational methods for SL prediction can be categorized into three types: statistical inference, network-based methods, and supervised machine learning methods (Wang et al. 2022a). The statistical methods are based on pre-defined hypotheses or rules to mine SL pairs. For instance, Jerby-Arnon et al. (2014) developed a data mining pipeline for SL inference named DAISY, assuming that two genes with SL relationship should be co-inactivated less than expected but co-expressed significantly more frequently in cancer cells. Yang et al. (2021a) proposed a model named SiLi to predict SL pairs in liver cancer, utilizing five inference strategies (i.e. functional similarity, differential gene expression, pairwise gene co-expression, pairwise survival, and rank aggregation). Network-based methods predict SL pairs by constructing biological networks and analyzing the topological features of genes in the networks. For example, Liu et al. (2018) simulated a human cancer signaling network and predicted SL pairs based on the distances between cancer genes and non-cancer genes in the network. Ku et al. (2020) used a clustering method to predict KRAS-related SL clusters from a protein–protein interaction (PPI) network and identified vital pathways for the clusters. Both types of methods have good interpretability since the hypotheses or network topologies can shed light on biological mechanisms underlying SL. However, the manual selection of hypotheses or topological features can be subjective, and these methods cannot make use of the known SL pairs identified by wet-lab experiments.

Another type of methods is supervised machine learning, which can be divided into traditional machine learning and deep learning methods. For instance, DiscoverSL uses multi-omics data (copy number alteration, gene expression, mutation, etc.) to extract gene features and employs random forest to predict SL pairs. SL^2^MF (Liu et al. 2020b) is a matrix factorization (MF) model which encodes the SL graph as a matrix and factorizes the matrix to learn the representations of gene pairs. Most traditional machine learning methods need to select gene features manually, which relies on prior knowledge and may cause biases. MF-based methods cannot fully explore the structures of an SL graph for predicting novel SL pairs. The deep learning methods mostly learn and combine the latent representations of two genes to predict if they have the SL relationship. DDGCN (Cai et al. 2020) is the first graph neural network model for predicting SL pairs, and it uses graph convolution network (GCN) and a dual-dropout mechanism to address the issue of data sparsity and overfitting. GCATSL (Long et al. 2021) is a graph attention network incorporating known SL pairs and gene features derived from the PPI network and gene ontology (GO). Although these methods have achieved good prediction accuracy, they are mostly black boxes lacking interpretability, i.e. offering little insight into biological mechanisms underlying the predicted SLs.

Incorporating prior knowledge into the above supervised learning models can improve their interpretability. Knowledge graph (KG) is a graph-based data model to integrate structured knowledge using a network of entities (nodes) and relations between entities (edges of various types), which is a type of heterogeneous graph. The nodes and edges in a KG usually have well-defined descriptions which are easy to understand (Ji et al. 2022). KGs (and heterogeneous graphs in general) have been popular in some application domains such as recommender systems, precision medicine, and drug discovery (Rotmensch et al. 2017; Chen et al. 2021; Zeng et al. 2022). By exploring the graph structures of gene pairs in a KG, the semantic relationships between genes as nodes in the KG can be captured to predict SL interactions. KG4SL (Wang et al. 2021) is the first method for SL prediction that is based on KGs. It uses a graph neural network (GNN) which samples the 1-hop neighbors of genes from a KG to capture the local graph structures around the genes. PiLSL (Liu et al. 2022) learns the representation for a pair of genes based on an enclosing subgraph extracted from a KG. SLGNN (Zhu et al. 2023) extracts shared factors (entity types) from a KG and designs a factor-based GNN model to learn gene representations.

However, the above KG-based methods could fail to find some structures or patterns in a KG that are important for the predictions and interpretability for the following reasons. First, KG4SL and PiLSL randomly sample the neighbors for a gene or a pair of genes, and thus some important semantic structures of the KG may be excluded when learning the gene representations. Second, these methods are based on gene embeddings, i.e. two genes with similar embeddings are likely to have SL relationship. As such, KG structures are implicitly encoded into the latent embeddings, which makes it hard to find important graph structures directly. Although PiLSL has used the learned attention weights to extract important subgraphs as explanations, the attention weights of different layers may lead to different explanations. SLGNN selects the important entity types as explanations, whereas such entity types cannot explain the specific SL mechanisms. Therefore, it is urgently needed to develop an explainable AI model that is able to capture the crucial KG structures for SL prediction and interpretation.

Path-based reasoning on KG has been widely used in recommendation systems, KG completion and drug-disease prediction (Wang et al. 2019a; Liu et al. 2021; Geng et al. 2022). A “relational path” connects two nodes in a KG through sequential relations (i.e. triples), which can be regarded as a rule to infer the relationship between the two nodes (Lao et al. 2011). As such, the path-based connectivity between a pair of nodes derived from KGs empowers the model to perform predictions and explanations. Recently, Zhang and Yao (2022) introduced a graph structure named “relational directed graph” (relational digraph for short), composed of overlapping relational paths, and they proposed a GNN-based method named RED-GNN for KG reasoning. Using dynamical programming, RED-GNN can efficiently extract and learn from relational digraphs for KG reasoning. Inspired by this study, we harness relational digraphs from a biomedical KG focused on SL to learn the semantic representations for SL predictions.

Here, we propose Knowledge Graph Reasoning for Synthetic Lethality (KR4SL), an end-to-end framework for explainable prediction of SL interactions. First, we design an encoder which automatically constructs multiple relational digraphs from a KG, and then performs reasoning within these graphs starting from the primary gene. Second, during the reasoning process at a layer, we combine the structure information of the relational digraphs and textual semantics of entities as propagated messages, and we further enhance the sequential semantics of the relational paths within each relational digraph. In this way, we can obtain powerful semantic representations for gene pairs to support SL predictions. Third, an attentive aggregation is conducted to distinguish the importance among different edges, so we can select the most important edges to form paths as explanations. Experimental results under two scenarios show that KR4SL achieves the best performance compared with all the baselines. By analyzing the explanations in two case studies, we find that our model can not only interpret the prediction process but also provide explanatory evidence for the predicted SLs consistent with biological prior knowledge. In summary, our contributions are as follows:

- We highlight the importance of deploying the structural information of relational digraphs from a heterogeneous graph for SL prediction.
- We present a novel KG reasoning-based model named KR4SL which learns the semantic representations of gene pairs by efficiently encoding the structural information of relational digraphs in the KG and the textual semantics of entities and enhancing the sequential semantics using a gated recurrent unit (GRU). In addition, it uses attention mechanisms to identify important subgraphs as explanations. In short, KR4SL integrates the strengths of path-based methods and GNN-based methods for KG reasoning and offers interpretability.
- We conduct experiments on a real dataset, under two different settings. The experimental results demonstrate that KR4SL has superior performance for SL prediction compared with multiple state-of-the-art models. In addition, through case studies, we show that KR4SL can provide intuitive explanations by revealing the prediction process and providing evidence that sheds light on biological mechanisms of SL.

## 2 Methods

In this section, we first briefly exhibit the preliminaries and problem formulation, and then we introduce the detailed implementations of our framework.

### 2.1 Problem statement

**SL graph**. We represent the observed SL interactions as a graph $G_{1}$, defined as a set of SL pairs $\left\{(g_{u},g_{v})|g_{u},g_{v}\in V\;\text{and}\;u,v\leq M\right\}$, here *M* is the number of genes and $V=\{g_{1},g_{2},\ldots ,g_{M}\}$ is a set containing all the genes in $G_{1}$.

**Knowledge graph**. In addition to the SL interactions, we also have external knowledge about the functions of genes, such as pathways and biological processes. We represent these information as a KG $G_{2}$, composed of a set of triples $\left\{(e_{h},r,e_{t})|e_{h},e_{t}\in E,r\in R\right\}$ where E is a set of entities and R is a set of relations. G is a directed graph, and each triple in $G_{2}$ represents a type of semantic meaning. For example, (TP53, involved_in, double-strand break repair) means that the gene TP53 is involved in the biological process of double-strand repair.

**Relational directed graph**. Suppose that we have a heterogeneous graph G, constructed by merging the known SL graph $G_{1}$ and KG $G_{2}$. To do this, we directly map genes from $G_{1}$ to entities in $G_{2}$, and we add new edges for corresponding gene pairs according to $G_{1}$. The new edges are bidirectional and represent SL relationships. Thus G is a directed graph. A “relational path” is defined as a sequence of relations in G, connecting a source entity and a target entity. The concept of “relational directed graph” is introduced by Zhang and Yao (2022). A relational directed graph (relational digraph) $G_{g_{q},g_{p}}^{K}$ is a graph in G with a source entity *g_q_* and a target entity *g_p_*. The graph has *K* layers, and the nodes at a same layer are different from each other. In this graph, any path from *g_q_* to *g_p_* is a relational path with length *K*, as a form of $g_{q}\overset{r^{1}}{\to }\cdot \overset{r^{2}}{\to }\cdots \overset{r^{K}}{\to }g_{p}$, here *r^k^* is an edge pointing from a node in the (k−1)-th layer to a node in the *k*-th layer.

**Problem statement**. Given a primary gene *g_q_* and the heterogeneous graph G, we assume that $V_{q}\subseteq V$ is the set of all known SL partners of *g_q_*, and $V_{q}^{u}=V-V_{q}-\{g_{q}\}$ is the set of genes having unknown SL relationships with *g_q_*. Our task is to infer *N* genes from $V_{q}^{u}$ which are mostly likely to be the SL partners for *g_q_*. To achieve this, we can cast all genes in V as candidate partners of *g_q_* and predict the scores for them. Then from the predicted scores, we select the scores of genes in $V_{q}^{u}$ and rank them in descending order to determine the top-N candidate SL partners.

### 2.2 Overview of KR4SL

**Figure 1.**
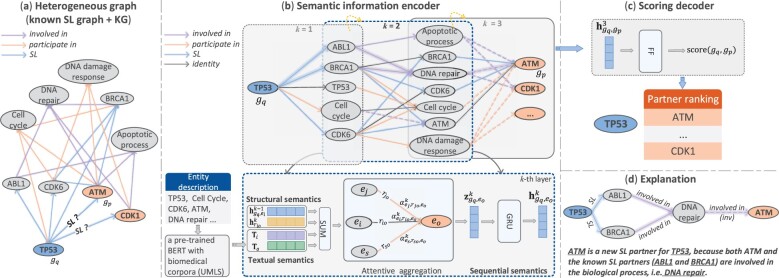
The Overall framework of our method. (a) A heterogeneous graph is constructed by a known SL graph and a KG, and our task is to predict novel SL partner genes (e.g. ATM and CDK1) for a primary gene (e.g. TP53). (b) The semantic information encoder of KR4SL. The top row is the union of 3-hop relational digraphs with the same source node TP53. Dashed arrows mean the reverse relations of corresponding colors. At each layer, the information is propagated from source entities to target entities. The bottom row is the detail of encoding process at the *k*-th layer. For each triple, the embeddings of textual semantics (extracted from a pre-trained BERT model) and embeddings of structural semantics are used to calculate the message, then attentive aggregation is performed among different triples with the same target. The sequential semantics is enhanced through a GRU and finally we get the semantic embedding for a target at this layer. (c) In the scoring decoder, the semantic embedding at the preceding layer is passed through a feed-forward layer to generate the score, measuring the possibility of a gene being a SL partner of TP53. All the candidates are ranked and top-N candidates are selected as the predicted SL partners of TP53. (d) For a predicted gene pair, the explanation subgraph is extracted from the relational digraph by choosing the edges with high attention weights learned during attentive aggregation.

In Fig. 1a, the known SL graph and the KG are combined as a heterogeneous graph G, which is a directed graph. We then augment the graph with reverse edges (having opposite directions with original edges) and identity edges (connecting nodes to themselves). Our model is composed of an encoder–decoder framework. As shown in Fig. 1b, for a primary gene *g_q_*, the designed encoder automatically finds relational paths and conducts reasoning along these paths. At each layer, the structural semantics of KG and the textual semantics (textual description of entities) are fused as the passed messages, and sequential semantics of relational paths are enhanced through a GRU module (Cho et al. 2014). We regard the genes at the last layer as the candidate SL partners of *g_q_*. In Fig.1c, The semantic representations of these candidates are passed through a scoring decoder to generate scores. We rank all the candidates according to the generated scores in descending order and select top-N candidates as the predicted partners for *g_q_*.

### 2.3 Semantic information representation encoder

Before diving into the details of our model, we introduce the overall reasoning procedures. For a primary gene *g_q_* (the initial source node) and one of its candidate gene *g_p_* (the target node), we repeat the following processes within $G_{g_{q},g_{p}}^{K}$ for *K* times:

- For a source node, find all the neighbors as the target nodes;
- Propagate the message from the source node to its target nodes and only calculate the representations of target nodes;
- Take target nodes as the source nodes for the next layer;

#### 2.3.1 Constructing relational digraphs for gene pairs with the same primary gene

Since the reasoning process is conducted within relational digraphs, we need to find the relational digraphs between a primary gene and all its candidate genes. A common approach is to extract subgraphs or paths for multiple gene pairs separately (Teru et al. 2020; Yang et al. 2021b; Liu et al. 2022). However, this may be time-consuming if there are many gene pairs in a large-scale graph. Here, we adopt an efficient method that can collectively derive relational digraphs between a primary gene and all its candidate gene (Zhang and Yao 2022).

Suppose that $G_{g_{q},e_{p_{1}}}^{K}$ is the *K*-hop relational digraph for $\left(g_{q},e_{p_{1}}\right)$, where all the relational paths have the length *K*. Similarly, $G_{g_{q},e_{p_{i}}}^{K}$ is the K-hop relational digraph for the source node *g_q_* and an arbitrary entity $e_{p_{i}}$. We can find that different relational digraphs share overlapping triples (refer to the exemplar graphs in Fig. 1b), thereby we can represent the union of all *K*-hop relational digraphs with source entity *g_q_* as where $\{e_{p_{1}},e_{p_{2}},\ldots ,e_{p_{N}}\}=E_{g_{q}}^{K}$ denotes all the entities at the *K*-th layer in $G_{g_{q}}^{K}$.


(1) $$G_{g_{q}}^{K}=G_{g_{q},e_{p_{1}}}^{K}\cup G_{g_{q},e_{p_{2}}}^{K}\cup \ldots \cup G_{g_{q},e_{p_{N}}}^{K},$$

Therefore, for the gene pairs with the same source gene *g_q_*, we do not need to extract the relational digraph. Instead, we can recursively construct the union of all K-hop relational digraphs $G_{g_{q}}^{K}$ for *g_q_*, using the strategy of dynamic programming (Xu et al. 2020). Specifically, we take the primary gene *g_q_* as the initial source node. Based on the graph $G_{g_{q}}^{k-1}$, we find all the neighbors at the *k*-th layer to obtain $G_{g_{q}}^{k}$. This process is similar to the breadth-first-search algorithm. At the *K*-th layer, we can get $G_{g_{q}}^{K}$ with a tree-like structure. The entities at the *K*-th layer are reachable nodes from *g_q_* within *K* steps, in which the genes are the candidate partners for *g_q_*.

#### 2.3.2 Message propagation along relational paths

Formally, starting from *g_q_*, we first construct $G_{g_{q}}^{k}$ based on $G_{g_{q}}^{k-1}$, then the information is propagated from the (k−1)-th layer to the *k*-th layer. Let $h_{g_{q},e_{i}}^{k-1}\in R^{d}$ be the semantic representation propagated from *g_q_* to *e_i_* with *k*−1 steps, where $e_{i}\in E_{g_{q}}^{k-1}$ is an entity at the (k−1)-th layer and *d* is the size of latent dimension. For a triple $\left(e_{i},r_{io},e_{o}\right)$ connecting the (k−1)-th layer with the *k*-th layer, we denote $h_{r_{io}}^{k}\in R^{d}$ as the representation of *r_io_* at the *k*-th layer, then we compute the semantic information passed from *e_i_* to *e_o_* as where $W_{1}^{k}\in R^{d\times d}$ is a linear transformation matrix at the *k*-th layer.


(2) $$h_{\left(e_{i},r_{io},e_{o}\right)}^{k}=W_{1}^{k}(h_{g_{q},e_{i}}^{k-1}+h_{r_{io}}^{k}),$$

Since semantic information is mainly learned from the KG. When the structure of the KG is very sparse, the reasoning process cannot be fully supported (Chen et al. 2020; Malaviya et al. 2020), and the model cannot provide informative interpretations. To enrich the semantics of KG for reasoning, we integrate the textual information into our model. The textual information is usually learned from large-scale unstructured corpora (e.g. articles), and it has been widely used in natural language processing (Liu et al. 2020a; Ju et al. 2022). Here, we take the entity descriptions in KG as the input and use a BERT model named CODER (Yuan et al. 2022) to generate textual embedding for each entity. Since CODER is pre-trained on a biomedical corpus (UMLS, which includes biomedical terms such as GO and gene), the textual embedding contains rich biological semantics related to our task. Suppose $T_{i}\in R^{d}$ is the textual embedding of *e_i_* and $T_{o}\in R^{d}$ is the textual embedding of *e_o_*, we modify Equation (2) as:


(3) $$h_{\left(e_{i},r_{io},e_{o}\right)}^{k}=W_{1}^{k}(h_{g_{q},e_{i}}^{k-1}+h_{r_{io}}^{k}+T_{i}+T_{o}).$$

In this way, the textual semantics and the structural semantics from KG are fused into the reasoning process.

We then generate the semantic representation for *e_o_*. Since there may be multiple source nodes connected with *e_o_*, to distinguish the importance of different nodes and the corresponding relations, we employ an attention mechanism to aggregate information from all source nodes (Veličković et al. 2018): where $W_{2}^{k}\in R^{d\times d}$ and $\alpha_{e_{i},r_{io},e_{o}}^{k}$ is the attention weight for the edge $\left(e_{i},r_{io},e_{o}\right)$ calculated by: where $W_{\alpha 1}^{k}\in R^{d}$ and $W_{\alpha 2}^{k}\in R^{d\times d}$ are two linear transformation matrices. $z_{g_{q},e_{o}}^{k}$ is the intermediate representation encoding information propagated from *g_q_* to *e_o_*.


(4) $$z_{g_{q},e_{o}}^{k}=\sigma \left(W_{2}^{k}\sum_{(e_{i},r_{io},e_{o})\in G_{g_{q},e_{o}}^{k}}\alpha_{e_{i},r_{io},e_{o}}^{k}h_{\left(e_{i},r_{io},e_{o}\right)}^{k}\right),$$

(5) $$\alpha_{e_{i},r_{io},e_{o}}^{k}=\text{Sigmoid}\left({\left(W_{\alpha 1}^{k}\right)}^{T}\text{ReLU}\left(W_{\alpha 2}^{k}h_{\left(e_{i},r_{io},e_{o}\right)}^{k}\right)\right),$$

#### 2.3.3 Learning sequential semantics

Next, we further enhance the sequential information from the (k−1)-th layer to the *k*-th layer, which can be also deemed to enhance the sequential dependencies of relational paths. We adopt a GRU (Cho et al. 2014) at the *k*-th layer to calculate the final semantic representation of *e_o_*: where $r^{k},f^{k},n^{k}$ are the reset, update, and new gates at the *k*-th layer, respectively. *σ* is the sigmoid function, and * is the Hadamard product. $W_{r}^{k},W_{f}^{k},W_{n}^{k},U_{r}^{k},U_{f}^{k},U_{n}^{k},b_{r}^{k},b_{f}^{k}$ and $b_{n}^{k}$ are learnable parameters.


(6) $$\begin{array}{cc} & r^{k}=\sigma \left(W_{r}^{k}z_{g_{q},e_{o}}^{k}+U_{r}^{k}h_{g_{q},e_{o}}^{k-1}+b_{r}^{k}\right),\\ & f^{k}=\sigma \left(W_{f}^{k}z_{g_{q},e_{o}}^{k}+U_{f}^{k}h_{g_{q},e_{o}}^{k-1}+b_{f}^{k}\right),\\ & n^{k}=\sigma \left(W_{n}^{k}z_{g_{q},e_{o}}^{k}+r^{k}(U_{n}^{k}*h_{g_{q},e_{o}}^{k-1})+b_{n}^{k}\right),\\ & h_{g_{q},e_{o}}^{k}=(1-f^{k})*n^{k}+f^{k}*h_{g_{q},e_{o}}^{k-1}, \end{array}$$

After *K* steps, we can obtain the representations of all entities at the *K*-th layer. From these entities we choose the genes to be the reachable candidate partners of *g_q_*. For instance, *g_p_* is one of the reachable candidate genes starting from *g_q_* with *K* steps, and $h_{g_{q},g_{p}}^{K}$ represents the semantic information propagated from *g_q_* to *g_p_*.

### 2.4 Scoring decoder

Given the representation for a primary gene *g_q_* and its candidate *g_p_*, we then calculate the scores for *g_p_*, indicating the possibility of gene *g_p_* becoming the SL partner of *g_q_*. We leverage a feed-forward layer as the scoring function: where $W_{ff}\in R^{d}$ and *b_ff_* are learnable parameters. Note that for those candidate genes that do not appear at the last layer, we set their scores as zeros. In this way, we can get the scores between *g_q_* and any genes in the SL graph.


(7) $$s(g_{q},g_{p})=W_{ff}^{T}h_{g_{q},g_{p}}^{K}+b_{ff},$$

### 2.5 Optimization

As we mentioned before, in our task, since all the genes in the SL graph can be the candidate SL partners for a primary gene, we regard each candidate partner as a class and use a multi-class loss function (i.e. cross-entropy) to train our model, through stochastic gradient descent strategy: where C is the set of gene pairs for training, and $C_{g_{q}}$ is particularly denoted as all the training gene pairs with the same primary gene *g_q_*.


(8) $$L=-\sum_{(g_{q},g_{p})\in C}\;\text{log}\;\frac{e^{s(g_{q},g_{p})}}{\sum_{\forall g\in C_{g_{q}}}e^{s(g_{q},g)}}$$

## 3 Results

We performed experiments on SynLethDB dataset under two scenarios, i.e., transductive setting and inductive setting (see more details in Section 3.1.2). Our empirical study aims to answer the following research questions:

- **RQ1**: How does KR4SL perform compared to the current start-of-the-art methods?
- **RQ2**: How does the modeling of semantic information affect KR4SL?
- **RQ3**: Can KR4SL provide explanations about predicting gene pairs, and are these explanations reasonable to reflect SL mechanisms?

### 3.1 Experimental setup

#### 3.1.1 Dataset description

The SL gene pairs we used are derived from SynLethDB-v2.0 (Wang et al. 2022b). The gene pairs in SynLethDB-v2.0 are collected from multiple sources, including wet-lab screenings (e.g. shRNA, RNAi, and CRISPR screenings), statistical screening (DAISY), text mining, etc. Additionally, a biomedical KG named SynLethKG is included in the database. Here, we focused on explaining SL pairs from the perspective of genetic functions. Thus, the knowledge about genes and their functions is extracted from the original SynLethKG. In the resulting KG, five types of entities include “Gene”, “Pathway”, and three types of GO terms, i.e. biological processes (BP), molecular functions (MF), and cellular components (CC). Four types of relations are included to describe if a gene is involved in a pathway or annotated with a GO term, namely, “Participate_in_PW, Participate_in_BP”, “Participate_in_MF”, and “Participate_in_CC”. Hence, the triples in this KG are all between a gene and other entities. To further enrich the semantic information of the KG, we complemented the knowledge about gene–GO and GO–GO, using the data from OntoProtein (Zhang et al. 2022). The final KG contains more types of relationships, and the overall statistics about the SL graph and the final KG are shown in Table 1. Details about the final KG are summarized in Supplementary Table S1.

**Table 1.** The statistics of the SL graph and the knowledge graph.

	# Entities	# Relations	# Triples
SL graph	9746	1	35,374
KG	42,547	32	38,1761

#### 3.1.2 Experimental settings

To fully evaluate the performance of our model, we conducted experiments under the following scenarios.

- Transductive reasoning: Given a SL graph and a KG, the model infers the missed SL partners (or SL interactions) for a gene to complement the SL graph. In this setting, we split the dataset by gene pairs, thus the genes in the test set may exist in the training set.
- Inductive reasoning: The model infers SL partners for genes unseen during training. In this setting, the training set and the test set have disjoint sets of genes. This setting could further examine the generalization ability of the model.

Recall that our model has no constraint on the number of negative gene pairs. During inference, for the primary gene, the model outputs the scores for all the candidate partner genes, and ranks the scores in descending order. Therefore, we leverage ranking metrics to evaluate the model performance, namely, NDCG@N (Normalized Discounted Cumulative Gain), Precision@N, and Recall@N (He et al. 2017; Wang et al. 2022c). Here, N is the number of candidate genes in the ranking list. The final metrics are averaged over all primary genes in the test set.

For transductive reasoning, we randomly split gene pairs as $T_{\text{train}},\;T_{\text{val}}$, and $T_{\text{test}}$ with the ratio of 7:1:2. 60% of $T_{\text{train}}$ are further sampled to be the known SL pairs. In this way, the KG and the known SL pairs are combined to construct the heterogeneous graph G, which is used to extract paths for training and inference. Note that all the undirected SL pairs are considered as bidirectional relations in our experiments.

For inductive reasoning, we follow the general setting of link prediction tasks to split the datasets (Teru et al. 2020; Zhang and Yao 2022). The set of SL genes is first divided into training genes and testing genes with the ratio of 6:4. Then, according to the two gene sets we obtained, we sample the corresponding subgraphs from the original KG: training sub-KG and testing sub-KG. The two sub-KGs have a disjoint set of genes, so that the genes used for testing are unseen during the training process. Next, for the SL pairs composed of training genes, we randomly split them as $K_{\text{train}},\;T_{\text{train}},\;T_{\text{val}}$ with the ratio of 4:3:3. $K_{\text{train}}$ is taken as the known SL pairs. We combine the training sub-KG with $K_{\text{train}}$ to construct $G_{\text{train}}$, which is used to be the input for $T_{\text{train}}$ and $T_{\text{val}}$. Similarly, for the SL pairs composed of testing genes, we generate $K_{\text{test}}$ and $T_{\text{test}}$ with the ratio of 4:6. $K_{\text{test}}$ and the testing sub-KG are used to construct $G_{\text{test}}$, which is the input for $T_{\text{test}}$. Same as in the transductive setting, all the SL pairs are augmented with reverse relations.

#### 3.1.3 Implementation details

In our model, we use three GNN layers, which means that all the relational paths have a length of three. The Adam algorithm (Kingma and Ba 2015) is used as the optimizer, and *L*_2_ regularization is adopted. We tune the weight decay rate in [$10^{-5},\;10^{-3}$] and the learning rate in [$10^{-4},\;10^{-3}$] with the maximum epoch 50. The batch size is set to 50, the latent embedding size is 48, and the dropout rate is 0.5. We run each experiment five times to compute the mean and standard deviation of results using an Nvidia Tesla V100 GPU.

#### 3.1.4 Baselines

We compared our proposed method with the following methods:

- DDGCN (Cai et al. 2020) designs a GCN model with dual dropouts to learn gene representations from the SL graph.
- SL^2^MF (Liu et al. 2020b) utilizes logistic MF to learn the gene similarities for SL prediction, and the learning process is constrained by GO and PPI annotations.
- SLMGAE (Hao et al. 2021) integrates the known SL graph, GO and PPI and designs a multi-view graph auto-encoder (GAE) to complete the SL interaction graph.
- GRSMF (Huang et al. 2019) reconstructs the SL interaction graph based on graph regularized self-representative MF. PPI and GO are utilized to regularize the learning process.
- GCATSL (Long et al. 2021) takes the known SL graph, PPI and GO as input graphs and incorporates a dual attention mechanism to complete the SL graph.
- KG4SL (Wang et al. 2021) adopts a GCN to learn the structural information of genes from a KG and predict SL interactions.
- SLGNN (Zhu et al. 2023) designs a factor-based GNN model to learn gene embeddings from a KG and a known SL graph.
- NSF4SL (Wang et al. 2022c) is the state-of-the-art method based on contrastive learning. The input features are pre-trained from a KG by TransE (Bordes et al. 2013), and two branches of neural networks are used to learn gene embeddings from SL pairs.

Among the above baselines, for fair comparisons, we use the same GO and PPI annotations for SL^2^MF, SLMGAE, GRSMF, and GCATSL. Note that PiLSL (Liu et al. 2022) also uses a KG for SL prediction. Different from KG4SL and our model, it requires extracting an enclosing graph from KG for each gene pair and then taking the enclosing graph as input. According to the setting of our task, all possible candidate genes of a primary gene are ranked according to their predicted scores. The extraction of the enclosing graphs for all possible gene pairs (∼40 million) is thus very time consuming. As such, we did not implement PiLSL as a baseline. In addition to the above baselines designed for SL prediction, we also implemented four methods designed for standard link prediction. ComplEx (Trouillon et al. 2016) and TransE (Bordes et al. 2013) are graph embedding methods, AnyBURL (Meilicke et al. 2019) is a rule-based method, HAN (Wang et al. 2019b) is a GNN model learning node embeddings from heterogeneous graph based on meta-paths.

### 3.2 Performance comparison (RQ1)

We conducted experiments under transductive and inductive settings. Tables 2 and 3 report the performance of various methods evaluated by seven ranking metrics. The complete results are reported in Supplementary Tables S2, S3, and S5. We categorized the baseline methods into two groups according to their input data. The first group of baselines (DDGCN, SL2MF, SLMGAE, GRSMF, and GCATSL) uses known SL pairs and gene features (GO and PPI) as input without using KGs, while the second group (KG4SL, SLGNN, and NSF4SL) take a KG as the input, in which SLGNN also makes use of known SL interactions.

**Table 2.** Performance comparison among various methods under transductive setting. Each experiment is repeated for five times to calculate the mean value and standard deviation. The best performance in each column is marked in bold.

	NDCG@10	NDCG@20	NDCG@50	Precision@20	Precision@50	Recall@20	Recall@50
DDGCN	0.0985 ± 0.0047	0.1109 ± 0.0019	0.1243 ± 0.0038	0.2419 ± 0.0012	0.3079 ± 0.0115	0.2419 ± 0.0012	0.3079 ± 0.0115
SL^2^MF	0.1314 ± 0.0071	0.1448 ± 0.0066	0.1566 ± 0.0066	0.1879 ± 0.0052	0.2240 ± 0.0053	0.1874 ± 0.0052	0.2238 ± 0.0053
SLMGAE	0.1614 ± 0.0018	0.1823 ± 0.0028	0.1973 ± 0.0027	0.2744 ± 0.0048	0.3256 ± 0.005	0.2732 ± 0.0048	0.3254 ± 0.0053
GRSMF	0.2208 ± 0.0001	0.2412 ± 0.0002	0.2504 ± 0.0001	0.3838 ± 0.0003	0.4110 ± 0.0001	0.3835 ± 0.0003	0.4109 ± 0.0001
GCATSL	0.2305 ± 0.0027	0.2454 ± 0.0020	0.2597 ± 0.0021	0.3990 ± 0.0025	0.4424 ± 0.0001	0.3989 ± 0.0025	0.4424 ± 0.0001
KG4SL	0.1848 ± 0.0019	0.1988 ± 0.0040	0.2120 ± 0.0042	0.3449 ± 0.0200	0.3895 ± 0.0196	0.3446 ± 0.0200	0.3895 ± 0.0196
SLGNN	0.1742 ± 0.0128	0.1954 ± 0.0144	0.2167 ± 0.0132	0.3633 ± 0.0269	0.4370 ± 0.0191	0.3624 ± 0.0267	0.4368 ± 0.0191
NSF4SL	0.2314 ± 0.0221	0.2499 ± 0.0213	0.2694 ± 0.0203	0.3895 ± 0.0116	0.4531 ± 0.0092	0.3886 ± 0.0116	0.4528 ± 0.0092
KR4SL	**0.4669 ± 0.0106**	**0.4804 ± 0.0103**	**0.4864 ± 0.0098**	**0.6013 ± 0.0047**	**0.6243 ± 0.0017**	**0.5675 ± 0.0047**	**0.6197 ± 0.0017**

**Table 3.** Performance comparison among various methods under inductive setting. Each experiment is repeated for five times to calculate the mean value and standard deviation. “–“ means that the value is zero, indicating the method is not able to make predictions. The best performance in each column is marked in bold.

	NDCG@10	NDCG@20	NDCG@50	Precision@20	Precision@50	Recall@20	Recall@50
DDGCN	0.0007 ± 0.0005	0.0009 ± 0.0006	0.0016 ± 0.0005	0.0015 ± 0.0009	0.0041 ± 0.0009	0.0015 ± 0.0009	0.0041 ± 0.0009
SL^2^MF	–	–	–	–	–	–	–
SLMGAE	0.0022 ± 0.0009	0.0082 ± 0.0020	0.0142 ± 0.0021	0.0249 ± 0.0058	0.0487 ± 0.0065	0.0054 ± 0.0023	0.0249 ± 0.0058
GRSMF	–	–	–	–	–	–	–
GCATSL	0.0006 ± 0.0005	0.0009 ± 0.0006	0.0018 ± 0.0007	0.0022 ± 0.0012	0.0060 ± 0.0016	0.0022 ± 0.0012	0.0060 ± 0.0016
KG4SL	0.0032 ± 0.0008	0.0060 ± 0.0013	0.0153 ± 0.0079	0.0159 ± 0.0052	0.0563 ± 0.0367	0.0155 ± 0.0053	0.0560 ± 0.0366
SLGNN	0.0213 ± 0.0058	0.0255 ± 0.0073	0.0316 ± 0.0094	0.0608 ± 0.0213	0.0865 ± 0.0327	0.0604 ± 0.0213	0.0862 ± 0.0326
NSF4SL	0.1786 ± 0.0065	0.1951 ± 0.0045	0.2125 ± 0.0047	0.2865 ± 0.0045	0.3466 ± 0.0105	0.2843 ± 0.0044	0.3460 ± 0.0105
KR4SL	**0.3610 ± 0.0113**	**0.3759 ± 0.0107**	**0.3897 ± 0.0107**	**0.5487 ± 0.0021**	**0.5958 ± 0.0033**	**0.5477 ± 0.0021**	**0.5956 ± 0.0033**

For transductive reasoning, both GCATSL and NSF4SL have good performance, indicating that the known SL pairs and KG help predict new SL pairs. For inductive reasoning, most baselines fail to predict SL pairs with unseen genes. Basically, these methods are based on gene embeddings learned by capturing the topology of the genes in the SL graph or KG. However, we cannot obtain accurate embeddings for those genes unseen in the training data, as their topology in the graph is unknown. Meanwhile, NSF4SL does not rely on the graph topology to make predictions, thereby it can maintain good prediction performance.

Compared with all the baselines, KR4SL consistently yields superior performance in two scenarios. For example, under transductive setting, KR4SL improves 78.1%, 35.47%, 34.54% over the best baseline (NSF4SL) in terms of NDCG@50, Precision@50, and Recall@50. This indicates that it is beneficial to consider multi-hop structures of the heterogeneous graph without sampling neighbors, and integrating the information of known SL pairs and the semantics of KG is helpful for SL reasoning. Moreover, since KR4SL infers SL partners by learning the relation rules rather than gene embeddings, our model still performs very well under inductive setting.

### 3.3 Model analysis for KR4SL (RQ2)

We conducted additional experiments to investigate the impact of semantic information on model performance. We first performed ablation studies with four types of model variants. Then we analysed how the size of directed relational digraphs and the dimension of latent embeddings affect the model performance.

#### 3.3.1 Effect of semantic information

We derived seven variants from the original model. Instead of using all the neighbors at each layer, KR4SL-16n randomly samples 16 neighbors to be the target entities for each source entity, and similar notions for KR4SL-32n, KR4SL-64n, and KR4SL-128n. Here, the mean and median of total number of neighbors per source entity are 33 and 12, respectively (the two values are rounded up to integers), and the standard deviation is 94.52. ${\text{KR}4\text{SL}}_{w/o\;\text{text}}$ is a variant without using textual embeddings in Equations (3) and (2) is used to calculate the propagated message. ${\text{KR}4\text{SL}}_{w/o\;\text{att}}$ is designed by removing the attention weights when performing aggregation in Equation (4). Additionally, we introduced a variant named ${\text{KR}4\text{SL}}_{w/o\;\text{gru}}$ by removing the GRU module in Equation (6) and we let $z_{g_{q},e_{o}}^{k}=h_{g_{q},e_{o}}^{k}$ at the *k*-th layer.

Table 4 shows the results evaluated by seven metrics under the transductive setting. The complete results are shown in Supplementary Table S4. We compared the results of these variants with the original KR4SL, and we summarized our observations as follows.

**Table 4.** Effect of semantic information, N@N, P@N, and R@N are short for NDCG@N, Precision@N, and Recall@N, respectively. The best performance in each column is marked in bold.

	N@10	N@20	N@50	P@20	P@50	R@20	R@50
KR4SL	**0.4669**	**0.4804**	**0.4864**	**0.6013**	**0.6243**	**0.5675**	**0.6197**
KR4SL-16n	0.2256	0.2343	0.2328	0.2913	0.3065	0.2741	0.3048
KR4SL-32n	0.2666	0.2695	0.2754	0.3317	0.3708	0.3208	0.3706
KR4SL-64n	0.2833	0.3042	0.3126	0.4160	0.4376	0.3947	0.4335
KR4SL-128n	0.2962	0.3145	0.3256	0.4263	0.4691	0.4074	0.4659
${\text{KR}4\text{SL}}_{w/o\;\text{text}}$	0.4525	0.4648	0.4723	0.5881	0.6143	0.5545	0.6097
${\text{KR}4\text{SL}}_{w/o\;\text{att}}$	0.4574	0.4718	0.4801	0.5873	0.6194	0.5538	0.6148
${\text{KR}4\text{SL}}_{w/o\;\text{gru}}$	0.3363	0.3535	0.3655	0.5057	0.5401	0.4848	0.5378

- Compared with KR4SL, the performance of four neighbor sampling variants decrease dramatically. These variants only use partial paths to propagate the information, and both the structural semantics of KG and the textual semantics are not fully leveraged. From KR4SL-16n to KR4SL-128n, the model performance increases with the increasing number of sampled neighbors, demonstrating that more semantic information is exploited. KR4SL performs the best since it fully utilizes all the neighbors at each layer without sampling.
-
${\text{KR}4\text{SL}}_{w/o\;\text{text}}$ underperforms KR4SL in terms of all the metrics. This result demonstrates that integrating the textual semantics of entities is valuable when reasoning SL pairs on KG.
-
${\text{KR}4\text{SL}}_{w/o\;\text{att}}$ underperforms KR4SL, suggesting that the attention mechanism is necessary to capture the critical structures of the heterogeneous graph.
- The performance of ${\text{KR}4\text{SL}}_{w/o\;\text{gru}}$ drops significantly compared with KR4SL, indicating that the sequential semantics learned by GRU is crucial for prediction. One reason could be that our model relies on the relational paths (acting as rules) to perform reasoning, and thus encoding the sequential dependencies of the relational paths would play important roles. Here, the GRU is able to capture sequential semantics, by memorizing the historical information along the relational paths and thereby enhancing the relational knowledge.

#### 3.3.2 Effect of the length of relational paths

**Figure 2.**
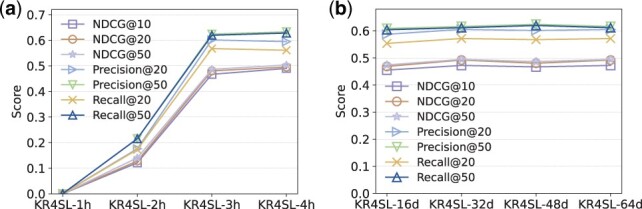
Parameter analyses: (a) effect of the length of relational paths and (b) effect of the dimension of latent embeddings.

To examine how the length of relational paths (i.e. the model depth) affects the model performance, we selected the number of GNN layers in the range of [1,2,3,4]. KR4SL-1h represents that the 1-hop neighboring genes of a primary gene are deemed as candidate partner genes, and similar notions for others. Figure 2a shows that increasing the model depth can boost the prediction performance. In particular, KR4SL-3h achieves a significant improvement compared with KR4SL-2h. Such results suggest that a directed relational digraph (or relational paths) should have at least 3-hop neighbors, so as to provide informative semantics for reasoning.

#### 3.3.3 Effect of the dimension of latent embeddings

To test how the dimension of latent embeddings affects the model performance, we selected the embedding dimension in the range of [16, 32, 48, 64]. KR4SL-16d means that the size of latent semantic embeddings *d* is set to 16, and similar notions for others. From Figure 2b, we can see that there is no obvious difference among all the models, except that the performance of KR4SL-16d is slightly lower than other models. This indicates our model is relatively stable when changing the size of the latent embeddings. Meanwhile, if the embedding size is too small, then the latent embeddings may not be informative enough to make good predictions.

### 3.4 Case study of explainability (RQ3)

Since KR4SL is to infer SL pairs along relational paths within relation digraphs, the whole reasoning process can be unraveled. Furthermore, benefiting from the attention mechanism, we can select the important paths (or subgraphs) from original relational digraphs for the predicted SL pairs. Such paths (or subgraphs) can provide the reasoning evidence and also reveal the corresponding SL mechanisms. Specifically, for a primary gene and one of its predicted partners, we first extracted the 3-hop relational digraph for the two genes. To find paths as complete as possible, for the first two layers, we chose the edges with attention weights higher than a threshold (which is set to 0.9 according to the distribution of attention weights). We further selected edges at the last layer with attention weights within the top five. As such, we can obtain the complete paths or a subgraph with multiple paths. Here we show two cases drawn from the learned KR4SL so as to demonstrate the explainability of KR4SL.

**Figure 3.**
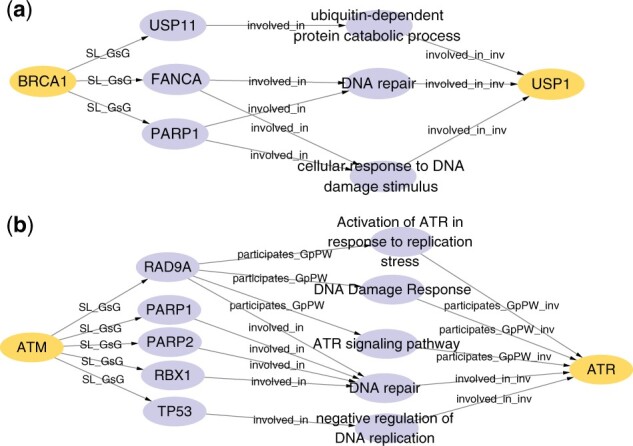
Explanations drawn from the learned KG4SL, which unveil the reasoning paths and evidence of inferring SL interactions. In each graph, the primary gene (source node) and the predicted partner (target node at the last layer) are highlighted. (a) Explanation subgraph for BRCA1 and USP1 and (b) explanation subgraph for ATR and ATM.

We first analysed the gene pair of BRCA1 and USP1. As a tumor suppressor gene, BRCA1 has an established role in DNA repair process. It can promote double-strand break repair through homologous recombination. USP1 is also crucial for DNA repair (Yoshida and Miki 2004). By controlling the PCNA monoubiquitination, USP1 is able to regulate the homologous recombination and repair the broken DNA strands (Huang et al. 2006). A previous study has shown that inhibiting USP1 can reduce the viability of BRCA1/2 mutant cancer cells (e.g. ovarian cancer cells), thereby USP1 is a SL partner of BRCA1 and has been a promising drug target in clinical cancer therapy (Simoneau et al. 2023). In our model, USP1 is predicted to be USP1 is the 25th SL partner among the top 50 candidates of BRCA1, and the top 50 candidates of BRCA1 are provided in Supplementary Table S6. In the explanation subgraph shown in Fig. 3a, we can see that there are multiple paths from BRCA1 to USP1, and these paths follow the same schema of BRCA1 $\overset{\text{SL}\_\text{GsG}}{\overset{}{\to }}$ a known SL partner of BRCA1 $\overset{\text{involved}\_\text{in}}{\overset{}{\to }}$ GO $\overset{\text{involved}\_\text{in}\_\text{inv}}{\overset{}{\to }}$ USP1. The schema can be cast as a rule which we used to reason the SL partners for BRCA1, and it shows that USP1 is a newly predicted partner for BRCA1 because USP1 and the known partner have GO terms in common. Specifically, two known SL partners (FANCA, PARP1) share the same biological processes (DNA repair and cellular response to DNA damage stimulus) with USP1. USP11 and USP1 are both involved in ubiquitin-dependent protein catabolic process. The three highlighted GO terms are closely related to the process of DNA repair, as we mentioned above. Therefore, the explanation provides reasonable evidence of why USP1 is responsible for the SL with BRCA1.

Another case is for ATM and ATR. ATR and ATM are signaling kinases that sense DNA damage and are major activators of DNA damage response (Kantidze et al. 2018). For ATM-defective cancer cells (e.g. CLL cells), ATR becomes the main activator to regulate DNA replication stress, and inhibition of ATR can induce unrepaired DNA damage and result in the cells death. ATR is a very promising target for treating ATM-defective tumors (Kwok et al. 2016; Wilson et al. 2022). Here, ATR is predicted to be the 34th SL partner among the top 50 candidates of ATM, and the top 50 candidates of ATM are reported in Supplementary Table S7. From the explanation subgraph in Fig. 3b, we observed a schema similar to that of BRCA1 and USP1, i.e. ATM $\overset{\text{SL}\_\text{GsG}}{\overset{}{\to }}$ a known SL partner of ATM $\overset{\text{involved}\_\text{in}}{\overset{}{\to }}$ GO $\overset{\text{involved}\_\text{in}\_\text{inv}}{\overset{}{\to }}$ ATR. Here, five function nodes (three pathways and two biological processes) are shared by ATR and known SL partners of ATM. These functions (especially Activation of ATR in response to replication stress and negative regulation of DNA replication) are closely related to the SL mechanism we mentioned above. Hence, they can be key evidence explaining why ATR and ATM have the SL relationship.

## 4 Conclusion and discussion

In this article, we proposed an interpretable deep learning pipeline named KR4SL to recommend novel SL partner genes for primary genes using KG reasoning. Using dynamical programming to explore the subgraph structures in the KG, our model efficiently extracts the relational digraphs for a primary and all its candidate partners from a heterogeneous graph composed of known SL gene pairs and a biomedical KG. Each path within a relational digraph consists of a sequence of relations (i.e. triples in the KG), representing a rule supporting the process of KG reasoning. To capture the semantic information of the input graph, we designed an encoder which integrates the structural semantics of the graph, the textural semantics of entities in the KG and the sequential semantics of the extracted relational paths. Moreover, attentive aggregation is performed to distinguish important graph subgraphs that have contributed to the SL prediction significantly and hence can perform the role of model explanation. Experimental results under two settings demonstrate the superior performance of KR4SL. The case studies of subgraphs show that our model is explainable since it not only uncovers the reasoning process for the SL prediction but also provides evidence that sheds light on potential SL mechanisms.

In the future, we plan to improve the interpretability based on this model. The extraction and interpretation of relational paths from the KG can be guided by biological prior knowledge (Fang 2014). For instance, reinforcement learning (Liu et al. 2021) with logical rules can be adopted to selectively generate the biologically meaningful paths, which satisfy some rules defined by the biological SL models in a process of KG reasoning constrained by prior knowledge. Besides, the SL interactions should be distinguished among different cell lines, as the SL relationships tend to occur in a context-specific manner. To this end, we plan to incorporate more biomedical data and prior knowledge into our model, e.g. multi-omics data and biological networks such as PPI, signaling, and metabolic networks.

## Supplementary Material

btad261_Supplementary_Data

## Data Availability

The data used in this study are available in the source code repository.
