# Correction to: KR4SL: knowledge graph reasoning for explainable prediction of synthetic lethality

**DOI:** 10.1093/bioinformatics/btad768

**Published:** 2023-12-24

This is a correction to: Ke Zhang, Min Wu, Yong Liu, Yimiao Feng, Jie Zheng, KR4SL: knowledge graph reasoning for explainable prediction of synthetic lethality, *Bioinformatics*, Volume 39, Issue Supplement_1, June 2023, Pages i158–i167, https://doi.org/10.1093/bioinformatics/btad261.

The originally published version of this manuscript omitted the following author affiliation for Ke Zhang: University of Chinese Academy of Sciences, Beijing 100049, China.

This error has been corrected.

