# Supplementary material for: KR4SL: knowledge graph reasoning for explainable prediction of synthetic lethality
Source: Bioinformatics. 2023 Jun 30;39(Suppl 1):i158–67. doi: 10.1093/bioinformatics/btad261 (PMC10311291; doi:10.1093/bioinformatics/btad261)
Supplement: btad261_Supplementary_Data [file btad261_supplementary_data.pdf]

Supplementary Materials of “KR4SL: knowledge graph reasoning for explainable prediction of synthetic lethality”

Ke Zhang [1,2], Min Wu [3], Yong Liu [4], Yimiao Feng [1,5] and Jie Zheng [1,6,*]

[1]School of Information Science and Technology, ShanghaiTech University, Shanghai, 201210, China

[2]Shanghai Institute of Microsystem and Information Technology, Chinese Academy of Sciences, Shanghai, 200050, China

[3]Institute for Infocomm Research, Agency for Science, Technology and Research (A*STAR), Singapore 138632, Singapore

[4]Nanyang Technological University, Singapore 639798, Singapore

[5]Lingang Laboratory, Shanghai, 201602, China

[6]Shanghai Engineering Research Center of Intelligent Vision and Imaging, ShanghaiTech University, Shanghai, 201210, China

To whom correspondence should be addressed. Tel: +86 (021) 20684861;
Email: zhengjie@shanghaitech.edu.cn

Table S1: Details about the relationships in the knowledge graph we constructed.

Type	# Triples
(gene, parpicipates_GpPW, Pathway)	41726
(gene, NOT|enables, molecular function)	233
(gene, NOT|involved_in, biological process)	376
(gene, NOT|is_active_in, cellular component)	3
(gene, NOT|located_in, cellular component)	109
(gene, NOT|part_of, cellular component)	134
(gene, is_active_in, cellular component)	8749
(gene, located_in, cellular component)	51382
(gene, part_of, cellular component)	60679
(gene, acts_upstream_of_or_within, biological process)	202
(gene, acts_upstream_of, biological process)	158
(gene, acts_upstream_of_negative_effect, biological process)	2
(gene, acts_upstream_of_or_within_negative_effect, biological process)	2
(gene, acts_upstream_of_or_within_positive_effect, biological process)	7
(gene, acts_upstream_of_positive_effect, biological process)	15
(gene, colocalizes_with, cellular component)	874
(gene, contributes_to, molecular function)	752
(gene, enables, molecular function)	54511
(gene, involved_in, biological process)	105623
(gene, NOT|colocalizes_with, cellular component)	9
(gene, NOT|contributes_to, molecular function)	3
(gene, NOT|acts_upstream_of_or_within, biological process)	1
(gene, NOT|acts_upstream_of_or_within_negative_effect, biological process)	1
(biological process, happens_during, biological process)	7
(biological process, has_part, biological process)	144
(biological process, has_part, molecular function)	94
(biological process, is_a, biological process)	33158
(biological process, negatively_regulates, biological process)	1858
(biological process, negatively_regulates, molecular function)	192
(biological process, occurs_in, cellular component)	119
(biological process, part_of, biological process)	3144
(biological process, positively_regulates, biological process)	1834
(biological process, positively_regulates, molecular function)	200
(biological process, regulates, biological process)	2092
(biological process, regulates, molecular function)	218
(cellular component, has_part, cellular component)	154
(cellular component, is_a, cellular component)	3224
(cellular component, part_of, cellular component)	1222
(molecular function, has_part, molecular function)	167
(molecular function, is_a, molecular function)	7493
(molecular function, negatively_regulates, molecular function)	50
(molecular function, occurs_in, cellular component)	31
(molecular function, part_of, biological process)	722
(molecular function, part_of, molecular function)	8
(molecular function, part_of, Pathway)	1
(molecular function, positively_regulates, molecular function)	40
(molecular function, regulates, biological process)	1
(molecular function, regulates, molecular function)	36

Table S2: Performance comparison among all methods under transductive setting. Each experiment is repeated for five times to calculate the mean value and standard deviation. N@N, P@N and R@N represent NDCG@N, Precision@N and Recall@N respectively. The best performance in each column is underlined.

	N@10	N@20	N@50	P@10	P@20	P@50	R@10	R@20	R@50
DDGCN	0.099±0.005	0.111±0.002	0.124±0.004	0.199±0.010	0.242±0.001	0.308±0.011	0.199±0.010	0.242±0.001	0.308±0.011
SL2MF	0.131±0.007	0.145±0.007	0.157±0.007	0.154±0.006	0.188±0.005	0.224±0.005	0.152±0.006	0.187±0.005	0.224±0.005
SLMGAE	0.161±0.002	0.182±0.003	0.197±0.003	0.216±0.002	0.274±0.005	0.326±0.005	0.213±0.002	0.273±0.005	0.325±0.005
GRSMF	0.221±0.000	0.241±0.000	0.250±0.000	0.327±0.000	0.384±0.000	0.411±0.000	0.326±0.000	0.383±0.000	0.411±0.000
GCATSL	0.231±0.003	0.245±0.002	0.260±0.002	0.356±0.003	0.399±0.002	0.442±0.000	0.355±0.003	0.399±0.002	0.442±0.000
KG4SL	0.185±0.002	0.199±0.004	0.212±0.004	0.301±0.011	0.345±0.020	0.389±0.019	0.300±0.011	0.345±0.020	0.389±0.019
SLGNN	0.174±0.013	0.195±0.014	0.217±0.013	0.296±0.021	0.363±0.027	0.437±0.019	0.294±0.021	0.362±0.027	0.437±0.019
NSF4SL	0.231±0.022	0.250±0.021	0.269±0.020	0.336±0.012	0.389±0.012	0.453±0.009	0.333±0.012	0.389±0.012	0.453±0.009
KR4SL	0.467±0.011	0.480±0.010	0.486±0.010	0.561±0.005	0.601±0.005	0.624±0.002	0.494±0.005	0.567±0.005	0.620±0.002

Table S3: Performance comparison among all methods under inductive setting. Each experiment is repeated for five times to calculate the mean value and standard deviation. '-' means that the value is zero, indicating the method is not able to make predictions. N@N, P@N and R@N represent NDCG@, Precision@ and Recall@N respectively. The best performance in each column is underlined.

	N@10	N@20	N@50	P@10	P@20	P@50	R@10	R@20	R@50
DDGCN	0.001±0.001	0.001±0.001	0.002±0.001	0.001±0.001	0.002±0.001	0.004±0.001	0.001±0.001	0.002±0.001	0.004±0.001
SL2MF	-	-	-	-	-	-	-	-	-
SLMGAE	0.002±0.001	0.008±0.002	0.014±0.002	0.005±0.002	0.025±0.006	0.049±0.007	0.005±0.002	0.025±0.006	0.049±0.007
GRSMF	-	-	-	-	-	-	-	-	-
GCATSL	0.001±0.000	0.001±0.001	0.002±0.001	0.001±0.001	0.002±0.001	0.006±0.002	0.001±0.001	0.002±0.001	0.006±0.002
KG4SL	0.003±0.001	0.006±0.001	0.015±0.008	0.006±0.001	0.016±0.005	0.056±0.037	0.006±0.001	0.016±0.005	0.056±0.037
SLGNN	0.021±0.006	0.025±0.007	0.032±0.009	0.046±0.015	0.061±0.021	0.086±0.033	0.045±0.015	0.060±0.021	0.086±0.033
NSF4SL	0.179±0.006	0.195±0.004	0.212±0.005	0.239±0.010	0.286±0.004	0.347±0.011	0.233±0.010	0.284±0.004	0.346±0.011
KR4SL	0.361±0.011	0.376±0.011	0.390±0.011	0.498±0.007	0.545±0.003	0.596±0.003	0.495±0.007	0.545±0.003	0.596±0.003

Table S4: Effect of semantic information. N@N, P@N and R@N represent NDCG@N, Precision@N and Recall@N respectively. The best performance in each column is underlined.

	N@10	N@20	N@50	P@10	P@20	P@50	R@10	R@20	R@50
KR4SL	0.467±0.011	0.480±0.010	0.486±0.010	0.561±0.005	0.601±0.005	0.624±0.002	0.494±0.005	0.567±0.005	0.620±0.002
KR4SL-16n	0.226±0.003	0.234±0.004	0.233±0.002	0.263±0.005	0.291±0.009	0.306±0.004	0.236±0.005	0.274±0.008	0.305±0.004
KR4SL-32n	0.267±0.004	0.269±0.002	0.275±0.001	0.313±0.009	0.332±0.003	0.371±0.000	0.288±0.008	0.321±0.003	0.371±0.000
KR4SL-64n	0.283±0.002	0.304±0.001	0.313±0.001	0.352±0.005	0.416±0.003	0.438±0.004	0.318±0.005	0.395±0.002	0.434±0.004
KR4SL-128n	0.296±0.004	0.314±0.002	0.326±0.003	0.356±0.007	0.426±0.003	0.469±0.001	0.312±0.006	0.407±0.002	0.466±0.001
$KR4SL_{w/o\ text}$	0.453±0.003	0.465±0.005	0.472±0.005	0.549±0.005	0.588±0.006	0.614±0.004	0.483±0.005	0.555±0.006	0.610±0.004
$KR4SL_{w/o\ att}$	0.457±0.007	0.472±0.013	0.480±0.010	0.541±0.020	0.587±0.023	0.619±0.008	0.475±0.021	0.554±0.023	0.615±0.008
$KR4SL_{w/o\ gru}$	0.336±0.021	0.353±0.019	0.365±0.013	0.446±0.011	0.506±0.011	0.540±0.002	0.406±0.007	0.485±0.010	0.538±0.002

Table S5: Performance comparison among different link prediction methods under transductive setting. Each experiment is repeated for five times to calculate the mean value and standard deviation. N@N, P@N and R@N represent NDCG@N, Precision@N and Recall@N respectively. The best performance in each column is underlined.

	N@10	N@20	N@50	P@10	P@20	P@50	R@10	R@20	R@50
TransE	0.049±0.000	0.059±0.000	0.075±0.000	0.074±0.001	0.107±0.000	0.167±0.000	0.073±0.001	0.107±0.000	0.167±0.000
ComplEx	0.084±0.000	0.099±0.000	0.117±0.001	0.142±0.001	0.187±0.001	0.252±0.001	0.141±0.000	0.187±0.001	0.252±0.001
HAN	0.135±0.005	0.154±0.007	0.171±0.006	0.199±0.014	0.264±0.023	0.338±0.018	0.199±0.014	0.264±0.023	0.338±0.018
AnyBURL	0.187±0.001	0.198±0.001	0.207±0.001	0.261±0.002	0.303±0.001	0.343±0.000	0.261±0.002	0.303±0.001	0.343±0.000
KR4SL	0.467±0.011	0.480±0.010	0.486±0.010	0.561±0.005	0.601±0.005	0.624±0.002	0.494±0.005	0.567±0.005	0.620±0.002

For ComplEx and TransE, we first extracted gene embeddings from the heterogeneous graph (composed of a KG and known SL data), and then we trained a multi-layer perceptron classifier to predict SL interactions. For the rule-based method of AnyBURL, we took the heterogeneous graph and the training SL pairs as input to mine rules, and then predicted SL interactions for the gene pairs on the test set. For HAN, we defined four meta-paths (i.e. Gene $\longrightarrow$ GO term $\longrightarrow$ Gene, Gene $\longrightarrow$ Pathway $\longrightarrow$ Gene, Gene $\longrightarrow$ a known SL partner $\longrightarrow$ GO term $\longrightarrow$ Gene and Gene $\longrightarrow$ a known SL partner $\longrightarrow$ Pathway $\longrightarrow$ Gene). Then, we fed our heterogeneous graph into HAN to learn gene embeddings. Finally, we calculated the prediction score for each pair of genes by taking the Hadamard product of the two gene embeddings and then adding the elements in the output vector together.

We can see that ComplEx and TransE do not perform as well as the other two methods, because they only consider the local graph structures. AnyBURL requires path sampling before extracting rules, which may lose some of the information needed for predictions. HAN performs significantly worse than KR4SL, probably because the learning process is constrained by the predefined meta-paths and the input graph for each meta-path is only sampled randomly, whereas KR4SL takes into account graph topological structures more thoroughly and systematically. As a result, the embeddings learned by HAN are not informative enough for accurate prediction of SL interactions.

Table S6: Top 50 candidate SL partners of BRCA1 predicted by KR4SL.

Rank	Gene	Rank	Gene	Rank	Gene	Rank	Gene	Rank	Gene
1	ATM	11	RAD51	21	MT1X	31	BCL2L1	41	WRN
2	RAD9A	12	RBX1	22	BAX	32	CHEK2	42	PML
3	ABL1	13	PMS2	23	PPM1D	33	ERBB2	43	ABCB1
4	BID	14	CETN2	24	RPL13A	34	CASP3	44	EP300
5	IGHMBP2	15	CDK6	25	USP1	35	CHEK1	45	TP53
6	POLM	16	RELA	26	PARP2	36	BCL2	46	CASP9
7	POLE	17	NHP2	27	RAD50	37	MAPK1	47	MYC
8	IGFBP3	18	RAD23B	28	AKT1	38	ATR	48	ABCA3
9	IGF1	19	KDR	29	H2AX	39	RAD21	49	XRCC6
10	WRAP53	20	BRCA2	30	SESN2	40	CDKN1A	50	SRC

Table S7: Top 50 candidate SL partners of ATM predicted by KR4SL.

Rank	Gene	Rank	Gene	Rank	Gene	Rank	Gene	Rank	Gene
1	MDM2	11	DNTT	21	MAPK12	31	RAD50	41	AKT1
2	ERCC1	12	RAD23B	22	POLD4	32	ERBB2	42	XRCC6
3	CDK2	13	MT1X	23	USP1	33	CASP3	43	MYC
4	H4C1	14	IGFBP3	24	H2AC8	34	ATR	44	BCL2
5	IGHMBP2	15	RAD51	25	SESN2	35	BRCA2	45	BLM
6	POLE	16	RPL13A	26	UNG	36	CASP8	46	PTEN
7	CETN2	17	POLD1	27	RRM1	37	PIK3CA	47	H2AX
8	NHP2	18	KDR	28	GTF2H1	38	BCL2L1	48	TP53
9	WRAP53	19	RELA	29	BRCA1	39	NAE1	49	BAX
10	ZBTB32	20	MTOR	30	ATM	40	BAK1	50	ERBB3
